# Demographic Correlates of Infant Feeding Practices and Growth Performance in the First Year of Life

**DOI:** 10.1155/2018/6569204

**Published:** 2018-10-01

**Authors:** Leila M. Shinn, Christy C. Tangney, Caitlyn Busche, Christine M. Sharp, Mary C. Mullen

**Affiliations:** ^1^Department of Clinical Nutrition, College of Health Sciences, Rush University Medical Center, Room 716, Armour Academic Center, 6000 S Paulina St., Chicago, IL 60612, USA; ^2^Supportive Oncology, Robert H. Lurie Comprehensive Cancer Center of Northwestern University, 250 East Superior Street, Suite 520, Chicago, IL 60611, USA

## Abstract

The aims of this study are (1) to assess changes in infant WHO growth indicators (weight-for-age, weight-for-length, and head circumference z-scores) from birth to 12 months of age as a function of feeding practices (FP) and (2) to describe the proportion of infants experiencing rapid weight gain (RWG; defined as change in weight-for-age z-score of ≥0.67 between birth and six months) among different FP. The modified Infant Feeding Practices Study II questionnaire was administered to 149 diverse caretakers/mothers of infants who were less than six months of age in a pediatric outpatient clinic. Growth as a function of FP was assessed using repeated measures ANOVA, while logistic regression was used to describe the correlates of RWG. The largest proportion of caretakers was African American (37%), 46% completed college, and 48% were enrolled in the Women, Infants, and Children (WIC) program. Regarding FP, 32% of infants were formula fed, and 18% were breastfed, with the remaining being either mixed fed or complementary fed, with nearly 40% of infants demonstrating RWG. While changes in weight-for-age z-scores differed among FP across time (p<0.05), observed patterns for head-circumference-for-age and weight-for-length z-scores did not. Various demographic correlates (caretaker race-ethnicity, education, and WIC enrollment) were associated with FP. Only the patterns of change in weight-for-age z-scores at 9 and 12 months differed among FP (with breastfeeding being the lowest at both time points). Further study is needed to adequately characterize the correlates of infant growth performance and growth patterns among different FP in such diverse samples. Continued research will allow for the development of an easy-to-use, succinct questionnaire that will allow healthcare providers to individualize feeding recommendations for caretakers of infants.

## 1. Introduction

Adequate nutrition during early life is crucial for proper growth, health, and development [1]. Malnutrition (both undernutrition and obesity) can have detrimental effects on a child's growth and cognitive development [2, 3]. According to the American Academy of Pediatrics (AAP) and the Academy of Nutrition and Dietetics (AND), exclusive breastfeeding during the first six months of life and continued breastfeeding during the first year of life, combined with the addition of complementary foods between four and six months of age, is beneficial for both the infant and mother [4, 5]. There is strong evidence that noncompliance to these guidelines may increase the risk of development of gastrointestinal and respiratory tract infections, otitis media, sudden infant death syndrome, and necrotizing enterocolitis in infants [6–10] and in addition may contribute to obesity. The impact of feeding practices (FP), especially breastfeeding on the development of obesity in later life, is less well understood [11–15]. In part, the lack of consensus is due to the varying infant ages when the presence of obesity is measured (2 or 6 years) and how obesity is operationalized (skinfold thickness or growth percentiles), the race-ethnicity of the infants studied, and the details of how feeding practice changes over time. The Infant Feeding Practices Study II (IFPS II) was developed to characterize US infant FP (breast, formula, mixed, and complementary feedings) used by caretakers throughout the infants' first year of life through the use of repeated questionnaires [16]. While a large sample of ~2000 pregnant women was tracked, the findings are limited by the fact that 84.4% of participants were non-Hispanic white, thus, limiting generalizability [17]. Considering this limitation, a modified version of the IFPS II (mIFPS II) questionnaire was developed, tested, and administered to women visiting urban pediatric outpatient clinics for primary care [18] to identify FP associated with infant growth performance in a more diverse population sample. While several groups have examined FP and growth through a variety of tools and measures, there is limited information on a diverse sample [19–21]. Moreover, researchers have not examined growth in a uniform manner; some utilize Center for Disease Control (CDC) and/or World Health Organization (WHO) growth charts [22–24].

The purpose of this descriptive study is to determine whether infant growth based on the WHO growth charts of a diverse group of infants varies across different infant FP reported using the mIFPS II questionnaire during the first six to 12 months of life. These findings can aid health care providers in developing nutrition-focused education strategies concerning feeding for mothers of infants from birth to six months of age.

## 2. Materials and Methods

### 2.1. Design, Setting, and Participants

We surveyed mothers/caretakers of infants about FP, demographics, and sources of nutrition information they accessed. These data were linked to longitudinal growth data for birth, one, three, six, nine, and 12 months. This was a convenience sample drawn from two urban outpatient pediatric clinics located at Rush University Medical Center in Chicago, IL.

Data on infant FP were collected at one time point between 28 days and six months of life through completion of the 34-item, paper mIFPS II questionnaire in which caretakers not only reported what their infant was being fed at that time, but also reported what FP they used while in the hospital and at time of hospital discharge. Caretakers had to be at least 17 years of age, able to speak and read English and have a 5^th^ grade or higher literacy level (measured by ability to comprehend consent form) or be willing to be interviewed by student researcher. Infants were excluded if they had any condition that prevented oral feeding or required parenteral or enteral feeding or any disease that affected growth patterns or oral feeding (e.g., cystic fibrosis, short bowel syndrome, cerebral palsy, and Down syndrome).

This study was approved by the Rush University Medical Center Institutional Review Board on June 3, 2016 (ORA #13042901). The mIFPS II questionnaires were administered to only those caretakers who gave informed consent. Caretaker and infant anonymity were preserved by use of study codes from the time of consent through all analyses.

### 2.2. Growth Data Collection

All available growth data were collected throughout the first year of life using the pediatric department's electronic medical record (EMR) (HYPERSPACE® Epic 2015). WHO growth standards were used to describe infant growth, specifically weight-for-age, weight-for-length, and head circumference z-scores. Infant measures were acquired and recorded into the EMR by health care providers (attending physicians, resident physicians, and nurses) in the urban pediatric outpatient clinic; data were converted into z-scores with software available in the EMR. All staff obtaining anthropometric measurements followed measurement recommendations provided by the American Academy of Pediatrics: A Bright Futures Handbook [25].

The analytic sample was restricted to any infant/caretaker dyad wherein at least two time points for weight, length, or head circumference were available in the EMR (first point being at birth and second point being at time of any subsequent visit ≤12 months of age). Rapid weight gain (RWG) was defined as weight gain z-score of greater than 0.67 from birth to six months of age [26]. The primary study variables included FP at time of questionnaire completion (exclusive breastfeeding, exclusive formula feeding, mixed feeding (a combination of breastfeeding and formula feeding) or complementary feeding (the introduction of solid foods in addition to one of the three aforementioned feeding practices), and growth measures. Mother's age (years), highest level of education completed, self-reported ethnicity, infant sex, and WIC enrollment were acquired from the mIFPS II questionnaire; prepregnancy body mass index (BMI) (kg/m^2^) was acquired from the EMR.

### 2.3. Statistical Analysis

The primary aim was to ascertain if infant FP are associated with growth changes in the first twelve months. Descriptive variables were summarized for the entire sample and compared between the four primary FP at the time of questionnaire administration. Chi-square tests were used to assess association between FP, education level, WIC enrollment, and race-ethnicity. ANOVA were used to assess differences in caretaker age, highest education attained, and prepregnancy BMI across FP.

There were two approaches to assess growth performance. These include rapid weight gain (RWG) over the first 6 months of life and the second approach, repeated measures of weight-for-age, weight-for-length, and head circumference-for-age z-scores during the first 12 months of life. Chi-square tests were conducted to assess the association between FP (breastfed, formula fed, mixed, or complementary) and rapid, moderate, and slow weight gain. Logistic regression with RWG as the outcome was conducted with FP as the predictor and additional demographic covariates (infant sex, and WIC enrollment). To test whether the growth patterns (weight-for-age, weight-for-length, and head circumference-for- age z-scores) differed across FP, we used repeated measures ANOVA with FP as the main effect, time (or infant age) and caretaker age, race-ethnicity, education, infant sex, and WIC enrollment as covariates. We controlled for covariates that we identified as potential confounders based on observed associations with FP or infant growth. These included caretaker age, caretaker race-ethnicity, caretaker education, WIC enrollment, infant birth weight, and prepregnancy BMI. Our basic model included caretaker age, education, and race-ethnicity. All statistical analyses were conducted using IBM SPSS Statistics for Windows, Version 23 (Armonk, NY) with a p-value of <0.05 as the level of significance.

## 3. Results

### 3.1. Sample Characteristics

As shown in Table 1, the average infant age of the entire sample (n=149) was 3.5 months, with 56% of all infants being <four months of age (n=83). However, this average increased to 6.1 months among infants who were complementary fed. Of the 37 breastfed infants, the largest proportion of caretakers was white (46%) and had a college degree or above (68%) with 16% enrolled in WIC. Nearly 42% of caretakers who were feeding formula to their infants were African American; equal proportions had some college education or a college degree or above (29% for both) and three-quarters were enrolled in WIC. African Americans and Hispanic caretakers equally comprised mixed feeders (33% for both) with 44% with a college degree or above and 33% enrolled in WIC. Finally, those infants who were complementary fed had caretakers who were 54% African American with 46% completing college or above and about half had been enrolled in WIC (54%). Chi-square analysis revealed that caretaker race-ethnicity, education level, and WIC enrollment were associated with FP (p<0.05 for all associations).

### 3.2. Growth Performance

**Figure 1:**
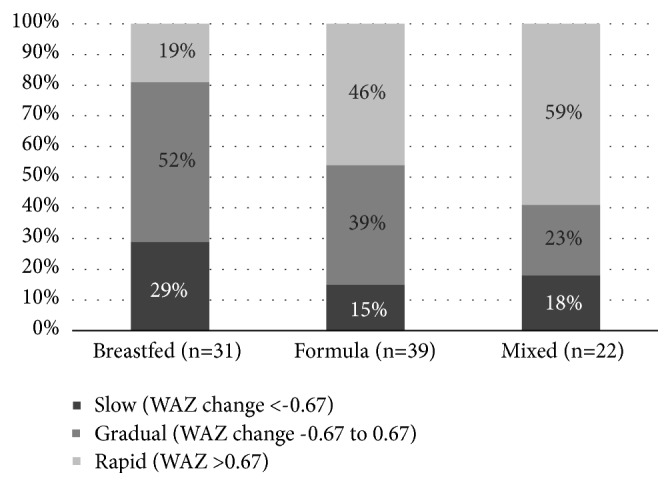
*Rate of weight gain between birth and 6 months of age for breastfed, formula fed, and mixed fed infants (n=92).* The rate of weight gain was calculated as weight-for-age z-score at 6 months of age minus weight-for-age z-score at birth. Values are percentages of infants in weight gain categories based on weight-for-age z-score change as slow (<-0.67), gradual (-0.67 to 0.67), or rapid (>0.67). We excluded the 35 infants who were complementary fed (and also previously fed by the alternate FP), there were 37 infants in total who experienced rapid weight gain by 6 months of age; again, FP and type of weight gain were related (*χ*^2^, p<0.05).

When examining rapid weight gain (RWG) as a dichotomous variable (yes/no), there were differences across feeding type (*χ*^2^, p=0.01). Of those with RWG, 13% were breastfed, 38% were formula fed, 28% were mixed fed, and 21% were complementary fed. When we excluded the 35 infants who were complementary fed (and thus previously fed by the alternate FP), there were 37 infants who experienced RWG by 6 months of age; again, FP and type of weight gain were related (p<0.05), as shown in Figure 1). With adjustment for infant sex and WIC participation, those who were mixed fed were almost five times more likely to have RWG at six months than those who were exclusively breastfed (adjusted odds ratio (aOR) = 4.70, p=0.01, 95% CI (1.44, 15.36)).

**Figure 2:**
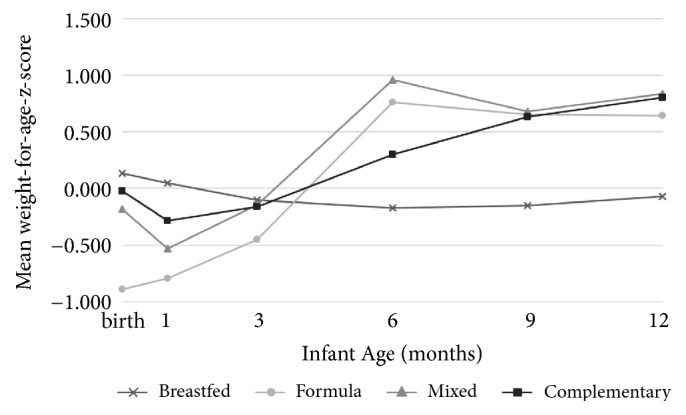
*Estimated mean weight-for-age z-scores at birth, 1 month, 3 months, 6 months, 9 months, and 12 months (n=60)*. Lines represent different infant feeding practices across time. Z-scores reflect adjustment for caretaker age, race, and education (p<0.05). There were some infants considered underweight (<5 percentile) at birth (8.3%), at 1 month (11.9%), at 3 months (6.3%), at 6 months (1.5%), 9 months (0%), and at 12 months (0%).

When weight gain was expressed as change in weight-for-age z-scores from birth to six months there were differences among the four FP (F=3.4, p=0.02); the magnitude of weight gain was lower among exclusively breastfed infants in comparison to those who were mixed fed (p<0.05, Bonferroni post hoc test) (data not shown). To examine growth patterns beyond infants at six months of age, we examined weight-for-age z-scores across time (Table 2). At both nine and 12 months, weight-for-age z-scores differed (p<0.05 and 0.021, respectively) among FP, with breastfeeding being the lowest at both time points (-0.035 and -0.040, respectively), followed by formula (0.360), then complementary feeding (0.610), and finally mixed feeding (0.894) at nine months and mixed (0.565) and then formula (0.600) and then complementary (0.780) at 12 months. These values are crude (unadjusted). When repeated measure models were run with race-ethnicity, education, and age of caretakers as covariates (additional models included WIC enrollment and caretaker prepregnancy BMI), changes in weight-for-age z-scores were the only growth parameter that exhibited differences among FP, p<0.05 (Figure 2); the estimated marginal means for weight-for-age z-scores among FP across the six time points are presented in this figure. No significant differences among infant FP were observed with weight-for-length or head circumference z-scores.

## 4. Discussion

In the present study, infant FP were described using the mIFPS II questionnaire. This questionnaire was designed to provide clinicians with information so that infant feeding recommendations and education based on growth performance could be individualized. Our long-term goal was to determine whether specific demographic characteristics altered the observed associations. Unlike many previous reports in the literature, more than one-third of this sample of caretakers/mothers were African American and nearly a quarter were Hispanic. Thus, this information was critically needed to inform the pediatricians and dietitians.

The evidence for the impact of different infant FPs on growth later in life has been inconsistent. Kavian et al. [26] examined rapid growth (weight gain z-score of ≥0.67 from birth to six months of age) and found that 36% of 670 Australian infants experienced RWG and those who were breastfed for less than four months were almost three times more likely to experience RWG (aOR=2.68 (95% CI (1.27-5.65))). Racial diversity was not reported in this large longitudinal study, nor was such reported in the Stockholm Weight Development Study [13]. While* duration* of breastfeeding was not queried in the present study because the mIFPS was only administered once within the first six months of the infant's life, 37% of our sample exhibited RWG (19% breastfed, 46% formula, 59% mixed, and 29% complementary), with RWG being most likely among infants who were mixed fed (aOR=4.70, p=0.01, 95% CI (1.44, 15.36)), using exclusive breastfeeding is the reference. This observation is suggestive of a protective benefit of exclusive breastfeeding.

Griffiths et al. [27] found that Caucasian infants who received no breast milk were more likely to exhibit a faster rate of weight gain from birth to 3 years of age than those who received any breastmilk (aOR=0.06, p<0.05, 95% CI (0.02 to 0.09)), regardless of duration and when adjusted for maternal social class, prepregnancy BMI, parity, smoking during pregnancy, and 3-year height z-score. Weight gain was also inversely related to breastfeeding duration; infants breastfed for less than four months were more likely to have RWG when compared to those breastfed for four months or more [27]. Again, these findings were based on a large sample of Caucasian infants, unlike the diverse sample in the present study. Kramer et al. examined the effects of various FP on growth through 12 months of age [28]. Similar to those of the present study, they found that mixed feeding and formula (or other milk) led to higher weight-for-age z-scores from three to six months of age when compared to exclusive breastfeeding.

In the present study, complementary feeding was associated with higher weight-for-age z-scores (p<0.05). In contrast, Kramer and coworkers found that cereal intake, which we could equate to complementary feeding, was associated with reductions in weight-for-age, length-for-age and head circumference z-scores [28]. It is important to note that Kramer et al. had a sample size of 17,046 in the Republic of Belarus, while the present study examined these z-scores in a much smaller, yet more diverse sample [28].

The present study is not without several limitations. First, questionnaire data were self-reported. Study sample size was limited and FP information was acquired at only one point in time with the majority of infants with survey data collected before complementary foods were initiated. Additionally, while a set protocol is followed to obtain infant measurements, these measurements were obtained at various appointments and therefore were subject to interrater variability. While we controlled for caretaker age, caretaker race-ethnicity, caretaker education, WIC enrollment, infant birth weight, and prepregnancy BMI in our statistical models, other potential confounding factors include gestational age and weight change during the first week of life. However, these variables were not routinely available and therefore were not controlled for. Finally, our convenience sample limits the generalizability of study findings.

## 5. Conclusion

In conclusion, infant FP was associated with race/ethnicity, caretaker education level, and WIC enrollment. The largest proportion of breastfeeding caretakers was white (46% versus 19% Hispanic), while most who formula fed their infants were African American (42%), followed by Hispanic (29%).

When comparing FPs with respect to RWG, formula fed (38% with RWG) and mixed fed (28% with RWG) not only had higher proportions of RWG, but also had higher median weight-for-age z-scores at nine and 12 months of age compared to those who were breastfed (13% with RWG) (p=0.02). Our findings indicate that growth trajectories differ by feeding practice at different time points throughout the first 12 months of life. However, further research is needed with larger diverse samples to verify these observations later in life.

Testing for test-retest reliability is needed. Further adjustments may be needed for our current version of the mIFPS II questionnaire. A more succinct and simplified questionnaire may also allow for the possibility of collecting feeding type at each growth measure visit (birth, one, three, six, nine, and 12 months) rather than just one static point in time.

## Figures and Tables

**Table 1:** Demographic characteristics of infants and caretakers by feeding type at time of questionnaire completion (n=149).

**Characteristic**	**Total Sample** (n=149)	**Breastfed** (n=37)	**Formula Fed** (n=48)	**Mixed** (n=27)	**Complementary** (n=37)
**Infant Agein ** **M** **o** **n** **t** **h** **s** ^*ז*^	3.5 (1.8, 4.9)	2.2 (1.6, 4.1)	2.3 (1.6, 4.1)	2.1 1.3, 4)	6.1 (6, 6.4)

**Caretaker Age in Years**	29.1 (24.9, 34)	30.7 (27.3, 34.2)	27.7 (24, 34.4)	28.6 (24.3, 32.9)	30.8 (25.2, 35.4)

**Male Infant **(n (%))	78 (52.3)	24 (64.9)	19 (39.6)	16 (59.3)	19 (51.4)

**Pre-pregnancy BMI**	25.3 (22.4, 31.1)	23.9 (22.5, 29)	25.9 (22.4, 29.8)	30.8 (26.6, 36.4)	25.3 (20.2, 31.5)

**Caretaker Race/** **E** **t** **h** **n** **i** **c** **i** **t** **y** ^*ז*^ (n (%))					
Non-Hispanic White	35 (23.5)	17 (45.9)	5 (10.4)	5 (18.5)	8 (21.6)
Non-Hispanic Black	55 (36.9)	6 (16.2)	20 (41.7)	9 (33.3)	20 (54.1)
Hispanic	35 (23.5)	7 (18.9)	14 (29.2)	9 (33.3)	5 (13.5)
Other	24 (16.1)	7 (18.9)	9 (18.8)	4 (14.8)	4 (10.8)

**Caretaker Education ** **L** **e** **v** **e** **l** ^*ז*^ (n (%))					
Less than 9^th^ Grade	1 (0.7)	0 (0)	0 (0)	0 (0)	1 (2.7)
9^th^-11^th^ Grade	12 (8.1)	1 (2.7)	7 (14.6)	4 (14.8)	0 (0)
High School/GED	21 (14.1)	3 (8.1)	12 (25)	2 (7.4)	4 (10.8)
Some College/AA	46 (30.9)	8 (21.6)	14 (29.2)	9 (33.3)	15 (40.5)
College Degree or Above	68 (45.6)	25 (67.6)	14 (29.2)	12 (44.4)	17 (45.9)

**WIC ** **E** **n** **r** **o** **l** **l** **m** **e** **n** **t** ^*ז*^ (n (%))	71 (47.7)	6 (16.2)	36 (75)	9 (33.3)	20 (54.1)

All values are expressed as median (IQR) unless otherwise specified. Missing data were as follows: for caretaker age, 2; for prepregnancy BMI, 88; for caretaker education, 1; and for WIC enrollment, 3.

^*ז*^Significant differences across feeding practices observed (p < 0.05).

**Table 2:** Weight-for-age z-scores by feeding type at the time of questionnaire completion.

	**Total Sample**	**Breastfeeding**	**Formula**	**Mixed**	**Complementary**
**Birth**	.060 (-.790, 0.625) (n= 145)	270 (-.475, .740) (n =37)	-.110 (-1.045, .310) (n =45)	.090 (-.890, .860) (n =27)	.145 (-.750, .595) (n =36)

**1 mo**	-.250 (-.908, .398) (n =118)	-0.600 (-.510, .675) (n =25)	-.430 (-.930, .180) (n =39)	-.035 (-1.228, .413) (n =26)	-.140 (-1.145, .470) (n =28)

**3 mo**	-.015 (-.723, .485) (n =144)	.040 (-.540, .240) (n =35)	-.150 (-.758, .295) (n =46)	.035 (-.748, .553) (n =26)	.110 (-.865, .660) (n =37)

**6 mo**	.200 (-.300, .860) (n =130)	-.030 (-.660, .470) (n =31)	.100 (-.335, .900) (n =41)	.245 (-.420, 1.413) (n =22)	.405 (-.105, .900) (n =36)

**9 ** **m** **o** ^*ɫ*^	.330 (-.200, 1.100) (n =107)	-.035 (-.695, .285) (n =26)	.360 (-.020, 1.120) (n =33)	.894 (-.200, 1.410) (n =19)	.610 (.215, 1.190) (n =29)

**12 ** **m** **o** ^*ɫ*^	.495 (-.040, 1.083) (n =80)	-.040 (-.620, .460) (n =19)	.600 (-.175, 1.075) (n =21)	.565 (.020, 1.538) (n =16)	.780 (.243, 1.273) (n =24)

All values expressed as median (IQR) and number of infants.

Growth data for the following time points include the following intervals: birth (0-0.2 months); 1 month (0.5-1.5 months); 3 months (2-4 months); 6 months (5-7 months); 9 months (8-10 months); 12 months (11-13 months).

^*ɫ*^Significant differences across z-scores stratified by infant feeding type at time of questionnaire completion (p<0.05). No other differences were observed based on Kruskal-Wallis tests.

## Data Availability

The data used to support the findings of this study are available from the corresponding author upon request.
